# Probable Cinnamon-Induced Mixed Hepatocellular-Cholestatic Liver Injury in a Young Woman: A Case Report

**DOI:** 10.7759/cureus.93328

**Published:** 2025-09-27

**Authors:** Sharoma J Yesukumar, Layaly Bakir, Ibrahim A Mohamed, Mohammed Danjuma

**Affiliations:** 1 Department of Medical Education, Hamad Medical Corporation, Doha, QAT; 2 Department of Internal Medicine, Hamad Medical Corporation, Doha, QAT

**Keywords:** cinnamon, drug-induced liver injury (dili), herb-induced liver injury (hili), herbs, liver, naranjo, r-factor, world health organization-uppsala monitoring centre (who-umc)

## Abstract

Reliable data on the safety of herbal medicines remains suboptimal. The consumption of these products is not without unanticipated harmful effects. Cinnamon, an herbal extract containing alkaloids such as coumarin, has been implicated in herb-induced liver injury (HILI). This report describes a rare case of HILI in a 34-year-old female, attributed to prolonged ingestion of cinnamon for weight loss purposes. She presented with abdominal pain, jaundice, and dark urine. Laboratory studies were consistent with acute liver injury. Cinnamon was immediately discontinued, and the patient was treated conservatively. On following visits, her liver biochemistry was back to normal with complete resolution of earlier features of hepatocellular injury.

## Introduction

Herbs and their products are widely used for various purposes, including the alternative management of chronic morbidities and improvement of general well-being [1,2]. In the US, a cross-sectional study involving more than 26,000 participants found that 35% of people use herbal supplements [1]. Although widespread, the efficacy and safety of these herbal products are not well studied in contrast to orthodox medicine. They can potentially cause undesirable side effects, particularly in the liver, where metabolism of these herbs occurs [2,3]. A segment of consumers who utilize these products operates under the presumption of their inherent safety compared to conventional pharmaceuticals, often remaining uninformed about their potential adverse effects [1-3].

Cinnamon is a widely used herbal supplement for the alternative management of diabetes and weight loss [4,5]. It has found value as “an anti-diabetic, anti-obesity, anti-inflammatory, and anti-tumor agent” for a cohort of the population who patronize alternative medicine [6]. Despite these presumed benefits, emerging reports have suggested cinnamon as a possible cause of herb-induced liver injury (HILI) [6-10]. This case report contributes novel evidence to the emerging literature on cinnamon-related HILI through its systematic application of validated causality assessment methodologies for adverse herb reactions.

## Case presentation

A 34-year-old woman presented to the emergency department with a one-week history of progressive epigastric pain radiating to the back. The pain was associated with nausea but was not accompanied by vomiting or diarrhea. It was exacerbated by food intake and initially responded to nonsteroidal anti-inflammatory drugs (NSAIDs). Three days before presentation, she noted dark discoloration of the urine, followed by the onset of scleral icterus one day before admission.

Her medical history was significant for gastroesophageal reflux disease and polycystic ovarian syndrome (PCOS), managed with metformin, which she has been taking without complications for several years. She denied any history of alcohol use. The patient reported a 10-year history of consuming cinnamon boiled in water several times weekly for weight loss. In the month prior to presentation, she had increased her cinnamon intake to twice daily. Her body mass index (BMI) was 30.

On examination, she was alert and in no acute distress. Mild scleral icterus was present, but there were no peripheral stigmata of chronic liver disease. Cardiovascular and respiratory examinations were unremarkable. Abdominal examination revealed a soft, nontender abdomen with no palpable organomegaly. There was no evidence of hepatic encephalopathy.

Initial laboratory investigations revealed a mixed pattern of transaminitis (Table 1). Comprehensive viral serological evaluation, including assays for hepatitis A, B, C, and E, Epstein-Barr virus, and cytomegalovirus, all yielded negative results. Ceruloplasmin, antinuclear antibody, anti-smooth muscle antibody, anti-liver and kidney microsomal antibodies, and iron studies (including serum iron, ferritin, transferrin saturation, and total iron-binding capacity) were within normal ranges.

**Table 1:** Laboratory results Hgb: hemoglobin, WBC: white blood cells, AST: aspartate transaminase, ALT: alanine transaminase, ALP: alkaline phosphatase

	On admission	Day 5	Day 13	Follow-up at one month	Normal range
Hgb	-	12	11.2	-	12-15 gm/dL
WBC	-	6	6.9	-	4-10 x 10^3^ uL
Platelets	-	280	289	-	150-410 x 10^3 ^uL
AST	329	346	24	11	0-32 U/L
ALT	506	699	94	10.6	0-33 U/L
ALP	261	202	119	87	35-104 U/L
Total Bilirubin	34	16	7	5.7	0-21 umol/L
Albumin	33	37	33	42	35-50 gm/L

Over the first five days of hospitalization, liver enzymes continued to rise, reaching a peak before gradually declining over the subsequent eight days. Throughout this period, the patient remained largely asymptomatic, aside from occasional abdominal discomfort. She was managed supportively with intravenous fluids and pantoprazole. All herbal supplements were discontinued, and she was counseled on dietary modifications. 

**Figure 1:**
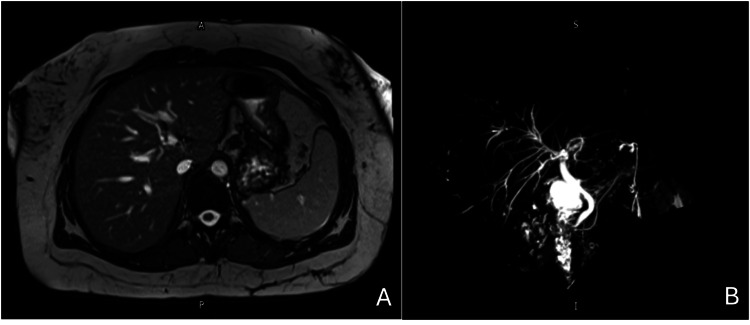
T2-weighted magnetic resonance image of the liver (A). Magnetic resonance cholangiopancreatography (MRCP) showing normal biliary tree (B).

A magnetic resonance cholangiopancreatography (MRCP) performed during her admission showed no evidence of biliary pathology (Figure 1). In addition, no evidence of malignancy was detected.

By the time of discharge, her clinical condition had significantly improved, with marked resolution of jaundice and abdominal pain. The patient’s management was limited to discontinuation of suspected hepatotoxic herbal agents, symptomatic care, and serial monitoring of hepatic biochemistry. During her follow-up clinic visit one month later, her liver enzymes were back to normal.

Case ascertainment

To systematically assess the likelihood of HILI in this patient, a structured adjudication approach was undertaken utilizing two well-validated adverse drug reaction (ADR) causality assessment tools: the Naranjo Probability Scale and the World Health Organization-Uppsala Monitoring Centre (WHO-UMC) system for standardized case causality assessment [11,12]. These tools, when used in combination, provide complementary quantitative and qualitative frameworks for ADR determination.

The patient’s score on the Naranjo scale was 6, corresponding to a "probable" ADR as follows: There were no previous conclusive reports on this reaction (0), the adverse events appeared after the suspected drug was given (+2), the adverse reaction improved when the drug was discontinued (+1), the drug was not readministered (0), there was no alternative causes that could have caused the reaction (+2), placebo was not given (0), drug levels were not done (0), the dose of the drug was not changed (0), reaction to previous exposure to the same drug is unknown (0), and the adverse event was confirmed by objective evidence (+1). 

This score reflects a plausible relationship between the onset of liver injury and exposure to the suspected herbal agents, in the absence of more likely alternative explanations. Concurrently, the WHO-UMC qualitative tool categorized the reaction as “probable/likely,” based on clinical judgment and the chronological relationship between exposure and injury, dechallenge response, and the exclusion of confounding etiologies.

To further characterize the pattern of liver injury, the R-factor for liver injury was calculated using the formula: R = (ALT ÷ ULN) / (ALP ÷ ULN). In this case, the R-factor was 10.9 on the fifth day of admission, consistent with a hepatocellular pattern of injury (R > 5) [13]. This pattern aligned with the patient’s clinical and laboratory findings and informed further workup to exclude competing causes, including viral and autoimmune hepatitis, and to evaluate liver morphology and biliary anatomy through imaging.

Taken together, the integration of these causality assessment tools and biochemical pattern analysis supported the probable diagnosis of HILI in this patient in the context of recent ingestion of cinnamon.

## Discussion

This case report provides evidence that cinnamon-infused water taken on an empty stomach daily for one month has likely caused this patient’s acute liver injury. This is supported by the very robust adjudication assessment framework highlighted above, as well as the historical details in the patient presentation. The patient's long-term use of metformin without prior hepatic issues makes it an improbable cause, particularly as the acute liver injury coincided with her increased cinnamon consumption rather than her stable medication regimen. In addition, the gradual and sustained improvement in her liver function due to cessation of cinnamon intake without any pharmacotherapeutic input further corroborates the clinical impression of HILI. On follow-up after one month, liver enzymes were notably within normal limits. Only symptomatic management was provided during her admission. The radiological diagnostics did not reveal any structural abnormality of the hepatobiliary tract, and she was negative for markers of hepatoviruses that may cause acute hepatitis. There was no evidence of malignancy on imaging studies. The PCOS was not documented by any imaging evidence, and she has been taking metformin for years without any suggestive symptomatology.

Several reports have documented adverse events associated with cinnamon supplementation in humans [9]. These reports show variability in the patient population, social demographic characteristics, and outcome measures. Brancheau et al. described hepatitis induced by concurrent use of cinnamon supplements and statins [7], a scenario distinct from our case, given our patient's lack of concomitant medications and our application of multiple standardized causality assessment tools for liver injury. Higaki et al. reported severe drug-induced liver injury (DILI) from an over-the-counter herbal preparation containing cinnamon and fennel [10], presenting partial parallels to our case. Although Higaki et al. employed the ADR adjudication tool DDW-J 2004 for DILI [10], the absence of comparative evaluation using established tools (WHO-UMC criteria, R-factor, and Naranjo scale) further distinguishes our methodological approach.

Despite growing interest in the development of HILI-related literature, data regarding cinnamon-induced liver injury remains limited. Previous studies have failed to combine adjudication tools such as the Naranjo, WHO-UMC, and R-factor to establish a robust association. This methodological limitation weakens the strength of causality attribution in existing reports. In addition, our patient presented with no confounding medications that could otherwise explain the observed hepatocellular injury, unlike prior studies that reported concurrent use of multiple herbal and pharmaceutical agents [8,10].

A thorough clinical history is critical when evaluating patients presenting with abdominal pain, jaundice, and dark-colored urine, particularly given the widespread use of herbal supplements. The duration and dosage of such supplements are key factors in identifying potential ADRs. However, quantification remains challenging due to the absence of standardized dosing regimens. Additional confounding variables must also be systematically excluded. 

This case report describes a patient who developed laboratory-confirmed hepatocellular injury after consuming cinnamon-infused water daily for one month. The close timing of symptom onset relative to increased cinnamon intake, the exclusion of alternative diagnoses, and the complete normalization of liver biochemistry following cinnamon discontinuation collectively support a probable causal relationship. These findings contribute novel evidence to the growing body of literature on HILI.

Strengths and limitations

The principal strength of this report lies in the application of multiple robust ADR adjudication tools to establish with reasonable certainty a case of cinnamon-related HILI. The Naranjo Adverse Drug Reaction Probability Scale yielded a score of 6, indicating a probable ADR, while the WHO-UMC causality assessment tool classified this suspected reaction as probable/likely related to cinnamon exposure. Notably, prior reports have not consistently applied this level of methodological rigor. Furthermore, our findings contribute to the accumulating evidence implicating dietary cinnamon exposure as a potential risk factor for liver injury.

As with all case reports, certain limitations must be acknowledged. These include uncertainty regarding the precise dosage and duration of metformin exposure in our patient, as well as the absence of mechanistic data definitively linking cinnamon to hepatotoxicity. In addition, we were unable to identify the specific cinnamon species consumed, which is an important consideration given the known variability in hepatotoxic potential among different *Cinnamomum* species. For instance, coumarin, the principal hepatotoxic constituent of cinnamon, is present in markedly different concentrations, ranging from <0.01 g/kg in *Cinnamomum verum* to 3.6 g/kg in *Cinnamomum cassia* (Cassia cinnamon) [9].

## Conclusions

This case report establishes a probable causal relationship between cinnamon exposure and HILI through the systematic application of multiple ADR causality assessment tools. The Naranjo scale and WHO-UMC causality assessment system collectively demonstrated a "probable" association. This comprehensive methodological approach, applied to a case without confounding polypharmacy, distinguishes our report from previous literature on cinnamon-associated hepatotoxicity.

The clinical course was characterized by acute liver injury following increased cinnamon intake. Comprehensive exclusion of alternative causes, including viral, autoimmune, and structural pathologies, further supports this association. The R-factor of 10.9 confirmed a hepatocellular injury pattern. The patient's complete biochemical and clinical recovery following cinnamon cessation, achieved through supportive management alone, strongly reinforces the diagnosis of HILI.

This case adds to the emerging evidence of cinnamon's hepatotoxic potential and highlights the importance of including herbal supplements in the differential diagnosis of acute liver injury. The widespread availability of these products, coupled with their common perception as safe alternatives to conventional medications, necessitates greater awareness among both clinicians and the public regarding their potential adverse effects.
